# Combining proteins with n-3 PUFAs (EPA + DHA) and their inflammation pro-resolution mediators for preservation of skeletal muscle mass

**DOI:** 10.1186/s13054-024-04803-8

**Published:** 2024-02-01

**Authors:** Renée Blaauw, Philip C. Calder, Robert G. Martindale, Mette M. Berger

**Affiliations:** 1 Division of Human Nutrition, Faculty of Medicine and Health Sciences, Stellenbosch University, Stellenbosch, South Africa; 2 Faculty of Medicine, University of Southampton, Southampton, UK; 3 NIHR Southampton Biomedical Research Centre, University Hospital Southampton NHS Foundation Trust, Southampton, UK; 4 Surgery Department, Oregon Health and Science University, Portland, OR USA; 5 Faculty of Biology and Medicine, Lausanne University, Lausanne, Switzerland

**Keywords:** Critical illness, Inflammation, Lean body mass, Nutrition, Protein

## Abstract

The optimal feeding strategy for critically ill patients is still debated, but feeding must be adapted to individual patient needs. Critically ill patients are at risk of muscle catabolism, leading to loss of muscle mass and its consequent clinical impacts. Timing of introduction of feeding and protein targets have been explored in recent trials. These suggest that “moderate” protein provision (maximum 1.2 g/kg/day) is best during the initial stages of illness. Unresolved inflammation may be a key factor in driving muscle catabolism. The omega-3 (n-3) fatty acids eicosapentaenoic acid (EPA) and docosahexaenoic acid (DHA) are substrates for synthesis of mediators termed specialized pro-resolving mediators or SPMs that actively resolve inflammation. There is evidence from other settings that high-dose oral EPA + DHA increases muscle protein synthesis, decreases muscle protein breakdown, and maintains muscle mass. SPMs may be responsible for some of these effects, especially upon muscle protein breakdown. Given these findings, provision of EPA and DHA as part of medical nutritional therapy in critically ill patients at risk of loss of muscle mass seems to be a strategy to prevent the persistence of inflammation and the related anabolic resistance and muscle loss.

## Introduction

Skeletal muscle is the largest tissue in the human body, accounting for approximately 40% of body mass in healthy adults [1]. Its metabolic and motor functions are threatened by injury and disease. Ageing and critical illness, with their associated inflammation, impair the healing capacity, and this is associated with poor global recovery and lower quality of life [2]. Critical illness results in an immediate and rapid loss of muscle mass varying between 17.7 and 21.8% by day 10 [3–5]. Such severe tissue loss is associated with an increased incidence of complications and ultimately death [6, 7]. In addition, patients are often admitted with pre-existing sarcopenia, an age-related (but not only) decline in muscle strength, quality, and mass [8], which further increases the risk of muscle catabolism compromising life perspectives.

In healthy adults, muscle mass preservation relies on a dynamic balance between muscle protein synthesis (MPS) and muscle protein breakdown (MPB) [9]. In critically ill patients, this balance is disturbed in favour of breakdown, with the extent of disturbance depending upon the severity of illness [10]. Excessive or unresolved inflammation promotes this imbalance: Inflammation inhibits the mammalian target of rapamycin (mTOR) signalling pathway that promotes MPS, and this contributes to the net muscle protein loss observed during critical illness and sepsis [11].

Inflammation is highly prevalent in critical illness [12] and triggers proteolysis within skeletal muscle [13, 14]. The aim of muscle catabolism is to mobilize amino acids for energy production (i.e. oxidation) or gluconeogenesis, with the ammonia released from the initial deamination being used for urea synthesis and excretion, and to support immunity, tissue repair and the production of acute-phase proteins [15]. However, after the initial acute phase, persistence of inflammation is deleterious, increasing the risk of ICU acquired weakness [16] and persistent critical illness [17], with its associated elevated urea-to-creatinine ratio, a signature of muscle catabolism [18]. Inflammation has two distinct phases, initiation and resolution: In persistent critical illness, this 2nd stage seems to be impaired.

How can this deleterious pathophysiological phenomenon be countered? Until recently, the principal strategies involved the delivery of adequate doses of proteins, higher proportions of essential and branched chain amino acids [19–21], and eventually physical activity [22]. The most recent strategy, which adds to the previous, is the use of very long-chain omega-3 polyunsaturated fatty acids (n-3 PUFAs) to drive resolution of inflammation and restore muscle health by activation of the resolution pathway [9]. These new findings are discussed herein.

### Role of proteins and amino acids

The current ESPEN ICU guidelines recommend a protein intake of 1.3-g protein equivalents/kg body weight/day [22], while ASPEN recommends 1.2–2.0 g/kg actual body weight/day [23]. Both ESPEN and ASPEN recommend using enteral nutrition (EN) as the first option to achieve energy and/or protein goals progressively over 48–72 h [22], and eventually to reach them within 3–7 days [23]. The ESICM guidelines share this strategy to start EN (when possible) within 48 h of admission at a low rate (10–20 ml/hour) and to build up slowly [24].

Despite the guidelines, the optimal protein dose and timing remain debated, as the impact on muscle protein breakdown is not clear. Wang et al. reported an inverse linear relationship between protein intake and 30-day mortality [25], while Zusman et al. observed mortality reduction with a protein intake exceeding 75% of target set at 1.3 g/kg/day [26]. However, others reported increased mortality with higher protein intakes [27, 28]. The EFFORT protein trial tested the prescription of different protein doses (> 2.2 g/kg versus ≤ 1.2 g/kg per day), ending with the mean delivery of 1.6 g/kg versus 0.9 g/kg per day in predominantly medical ICU patients: A major heterogeneity within groups was present, with an important overlap between groups, as well as a likely overfeeding in the intervention group with many patients receiving > 30 kcal/kg/day in the early phase [27]. Global outcome (discharge and mortality) did not differ between groups (i.e. null finding): The limitations make it difficult to conclude on the real impact of proteins, only that high-dose proteins are probably deleterious in the presence of acute kidney injury. The FRANS study which was conducted in 2015 under the previous guidelines confirmed that early full feeding was associated with worse outcome [28]: This involves both energy and protein delivery, but it is more the early pushing of nutrition which seems to have been the problem again not enabling to really conclude about the impact of protein dose. In the secondary analysis of the cluster randomized trial “Actively implementing an evidence based feeding guideline for critically ill patients” (NEED) data, the best probability for survival was reported for the group with medium intake of protein (average of 0.8 ± 0.18 g/kg/day) while the group with the highest protein intake (average 1.68 ± 0.39 g/kg/day) had the highest 28-day mortality risk compared to the medium intake group (hazard ratio of 2.32) [29]. The notion that a moderate protein intake is associated with the best outcomes was also supported with the results of the EuroPN study, where moderate protein intakes (0.8–1.2 g/kg/day) compared to both lower protein intakes (< 0.8 g/kg/d) or higher intakes (> 1.2 g/kg/day) were associated with earlier successful weaning (hazard ratio of 2.6) on day 12 [30]. It was also confirmed with a meta-analysis of 19 RCTs comparing higher to lower protein intake (and similar energy intakes), that the higher protein intake groups (1.31 g/kg/day) compared to the lower (0.9 g/kg/day) had no significant difference in mortality, length of stay, length of mechanical ventilation, or infections complications [31]. A progressive protein intake was proposed by Koekkoek et al. based on observing the lowest mortality with a protein intake on days 1–2 of less than 0.8 g/kg/day; followed by 0.8–1.2 g/kg/day on days 3–5 and more than 1.2 g/kg/day from day 5 onwards [32]. It is important to recognize that there are indications of potential harm with increased protein intake in the presence of multiple organ failures, especially acute kidney injury [27]: Of note, this negative effect resolved when dialysis was considered.

Anabolic resistance is defined as the inability of muscle to maintain its protein mass by appropriate stimulation of synthesis and inhibition of breakdown [33, 34]. It is favoured by immobility and inflammation and is observed in the critically ill and with ageing. Baseline inflammatory status was recently shown to affect response to nutrition therapy in medical patients: Based on admission C-reactive protein (CRP) level, nutritional management (see Swiss EFFORT trial [35]) resulted in a 66% mortality risk reduction in the group with low levels of inflammation (CRP < 10 mg/L) and a 59% mortality risk reduction in the group with moderate levels of inflammation (CRP 10–100 mg/L). However, the group with high inflammatory levels (CRP > 100 mg/L) had a 32% increased mortality risk after receiving medical nutrition therapy [36]. Similarly, the presence of sepsis affects mortality outcome after adequate protein intake where two studies have shown that the mortality benefit of protein intake of 1.2 g/kg/day on days 2–4 was lost in the presence of sepsis [6, 37].

Older individuals require higher protein intakes to overcome the anabolic resistance that occurs with ageing: The myofibrillar fractional synthesis rate is significantly higher at lower protein intake levels in younger individuals [38]. Nitrogen balance studies show similar results: Response to protein intake in ICU patients with trauma improved linearly up until an intake of 1.49 g/kg/day in younger patients, while a blunted effect was observed in the elderly group until an intake exceeding 1.5 g/kg/day [39].

Through measuring myofibrillar protein synthesis rates, Chapple et al. compared ICU patients to healthy controls and were able to show that there was no difference in protein digestion and amino acid availability post meal. However, muscle protein phenylalanine enrichment (as a measure of dietary protein incorporation into skeletal muscle protein) was 60% lower in the critically ill patients, which speaks to anabolic resistance in the critically ill [33], probably mediated through the persistent inflammation.

**Fig. 1:**
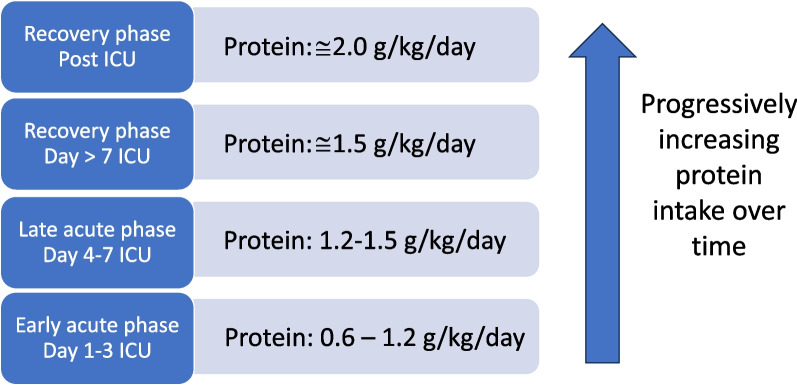
Schematic overview of protein progression strategy over time during a patient’s ICU stay. While the patient is in the acute phase, it is recommended that 1.5 g/kg/day protein (which equates to 1.2–1.3 g/kg/day protein equivalents) not be exceeded. The intake can be increased during the recovery and post-ICU phases, taking disease-specific guidelines into consideration

Protein requirements vary between individuals as there are many variables that influence response to protein intake. In the initial stages of critical illness, a gradual increase in protein intake of 0.6–1.2 g/kg/day is recommended, to be further progressively increased to 1.5 g/kg/day over the next few days provided stability is present (Fig. 1). When using parenteral nutrition, i.e. amino acids, only 83 g are produced from 100-g protein hydrolysate: Clinicians should compensate for the difference when aiming to achieve protein equivalents [40]. With a protein delivery adequacy compared to prescription of around 46–65% [30], it is crucial to ensure that actual protein targets are being met. Combining an adequate protein intake with early mobilization also results in less ICU-acquired weakness [41], significantly less muscle volume loss [42, 43], and lower mortality [41].

Balanced nutrition including protein is important. Feed early and progressively increase protein to target 1.3 g/kg/day protein equivalents over 4–7 days according to ESPEN ICU guidelines. Aim for protein adequacy of 70–100% of requirements. Adding n-3s (EPA + DHA) may support muscle protein metabolism.

## Inflammation, EPA, DHA, and pro-resolving mediators

**Fig. 2:**
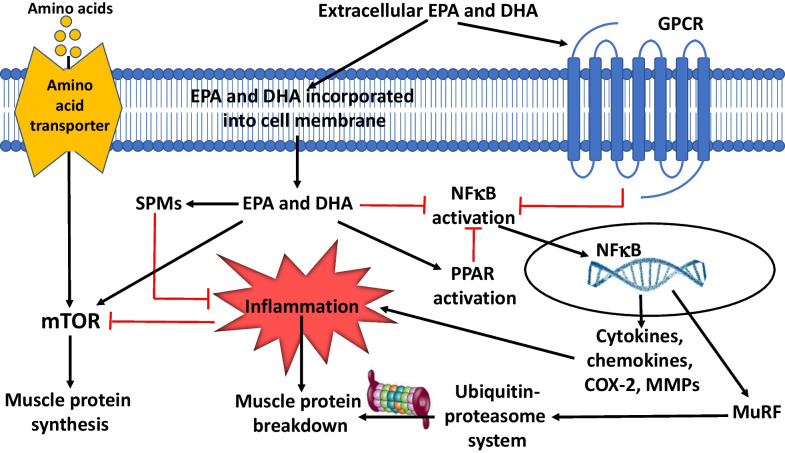
Cellular mechanisms by which EPA and DHA impact muscle protein synthesis and breakdown. Amino acids promote muscle protein synthesis via mammalian target of rapamycin (mTOR). Inflammation promotes muscle protein breakdown, partly through upregulation of the ubiquitin–proteasome system; inflammation also decreases protein synthesis through inhibition of mTOR. EPA and DHA have multiple anti-inflammatory actions and also promote inflammation resolution. Extracellular EPA and DHA are incorporated into muscle cell membrane phospholipids from where they may be released and act as substrates for specialized pro-resolution mediators (SPMs). Extracellular EPA and DHA can act as ligands for G-protein coupled receptors (GPCRs) especially GPCR120. Subsequent signalling inhibits activation of the pro-inflammatory transcription factor nuclear factor kappa B (NF*κ*B). EPA and DHA also inhibit NF*κ*B activation, probably via membrane-mediated actions, and they activate peroxisome proliferator-activated receptors (PPARs), which physically interfere with NF*κ*B translocation to the nucleus. NF*κ*B upregulates synthesis of genes encoding many proteins involved in the inflammatory response including multiple cytokines, chemokines, cyclooxygenase (COX)-2, and matrix metalloproteinases (MMPs) and upregulates muscle ring finger protein (MuRF) which activates the ubiquitin–proteasome system. Hence, the anti-inflammatory and inflammation resolving actions of EPA and DHA act to promote muscle protein synthesis and to decrease muscle protein breakdown. There is also evidence that EPA and DHA activate the mTOR pathway

Unresolved inflammation may be a key factor in driving persistent muscle catabolism. Inflammation is now recognized to have two distinct phases, initiation and resolution, both being required to mount an appropriate immune and healing response. The very long-chain n-3 PUFAs eicosapentaenoic acid (EPA; 20:5n-3) and docosahexaenoic acid (DHA; 22:6n-3) act through multiple interacting mechanisms to reduce inflammation [44]. The anti-inflammatory actions of EPA and DHA include decreased production of pro-inflammatory eicosanoids from arachidonic acid [45] and decreased activation of the pro-inflammatory transcription factor NF-*κ*B resulting in reduced expression of genes encoding pro-inflammatory cytokines, chemokines, and enzymes [45, 46]. EPA and DHA also increase production of interleukin (IL)-10 [47], an anti-inflammatory cytokine. By both reducing inflammation and promoting its resolution (Fig. 2), EPA and DHA might decrease muscle proteolysis and loss [48].

**Fig. 3:**
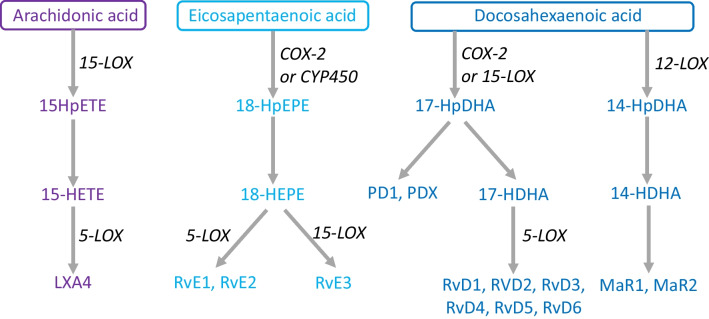
Metabolic pathways by which PUFAs (arachidonic acid, EPA, and DHA) give rise to downstream metabolites of inflammation resolution. Abbreviations used: COX, cyclooxygenase; CYP450, cytochrome P450; HDHA, hydroxy-DHA; HEPE, hydroxy-EPA; HETE, hydroxy-eicosatetraenoic acid; HpDHA, hydroperoxy-DHA; HpEPE, hydroperoxy-EPA; HpETE, hydroperoxy-eicosatetraenoic acid; LOX, lipoxygenase; LX, lipoxin; MaR, maresin; P, protectin; and Rv, resolvin

Until recently, it was believed that the resolution phase of inflammation was a passive process with inflammation declining when pro-inflammatory mediators diffused away from the area of injury. Now, based on elaborate studies conducted mainly by the Serhan group [49], we know that resolution is a biosynthetically active process that is initiated by compounds endogenously synthesized from EPA and DHA; these compounds are referred to as resolvins, maresins, and protectins [50, 51] (Fig. 3), and they are collectively named specialized pro-resolving mediators (SPMs). SPMs are also produced from the n-6 PUFA arachidonic acid (lipoxins) and from both n-6 and n-3 docosapentaenoic acid (DPA). When inflammation is initiated by any cause, it can follow one of the two pathways. The inflammation can continue unregulated and become chronic, resulting in ongoing tissue damage, persistent infection, and/or autoimmune disease. Alternatively, when present, SPMs induce a highly orchestrated inflammation resolution process, allowing for tissue repair and healing and ultimately a return to homeostasis [50].

SPMs are not present in the diet, rather they are produced endogenously via the respective metabolism of arachidonic acid, EPA, DPA, and DHA into a wide-ranging series of fatty acid derivatives; examples of the most commonly studied include lipoxin A4 (from arachidonic acid), resolvin E1 (from EPA), and resolvins D1 and D2 (from DHA). Lipoxin A4 produces its anti-inflammatory effects through induction of monocytes and macrophages to increase phagocytosis and stimulation of IL-10 production with reduction of pro-inflammatory cytokine release. Resolvin E1 decreases neutrophil activation and endothelial adhesion, while also decreasing production of reactive oxygen species. The SPMs also enhance microbial killing and clearance. Resolvin D1 acts upon endothelial cells by increasing intracellular nitric oxide and prostacyclin concentrations and decreasing adhesion receptors, ROS generation, and pro-inflammatory cytokines. Resolvin D2 decreases migration of dendritic cells and IL-12 production [51]. In summary, SPMs work to inhibit further neutrophil recruitment, promote secretion of pro-resolution cytokines with attenuation of pro-inflammatory cytokines, and increase clearance of microbes and cellular debris. In humans, fish oils are an important source of EPA and DHA. Supplementation of EPA and DHA has been found to increase circulating levels of many SPMs [52]. It has been shown that SPM synthesis is evolutionarily conserved: mice, frogs, and amoeba all synthesize SPMs showing the importance of inflammation resolution in tissue repair and recovery across nature [53]. The concentration range for activity of SPMs is in the nanomolar/picomolar level. They are found at bioactive levels throughout most tissues, including the brain, lymph nodes, and adipose tissue [49–51, 54].

To enable the beneficial muscle effects, EPA and DHA would need to be incorporated into skeletal muscle. Several human trials demonstrate increases in both EPA and DHA in skeletal muscle when their oral intake is increased. Andersson et al. [55] reported the effect of giving healthy participants aged 30–65 years a supplement providing 1.5-g EPA plus 0.9-g DHA daily for 3 months on skeletal muscle (vastus lateralis) phospholipid fatty acids. EPA was about 5 times higher than in the control group (~ 5% of fatty acids vs ~ 1% of fatty acids) while DHA was about 2 times higher (~ 4.2% of fatty acids vs ~ 2% of fatty acids). Smith et al. [56] reported that 1.86-g EPA and 1.5-g DHA taken daily for 8 weeks increased muscle (quadriceps femoris) EPA from 0.5 to 2.2% of fatty acids and DHA from 2 to 3.5% of fatty acids. These increases in EPA and DHA show a time dependency. McGlory et al. [57] took serial muscle (vastus lateralis) biopsies from young males supplemented with 3.55-g EPA plus 0.9-g DHA daily for 4 weeks. They reported increased EPA within 1 week and a progressive increase at 2 and 4 weeks with EPA increasing from 0.5 to 2.25% of fatty acids. DHA did not increase until 2 weeks and reached ~ 2% of fatty acids at 4 weeks, compared to the starting value of 1.5%. The slower incorporation of DHA than EPA into skeletal muscle is consistent with observations made for blood lipids, white blood cells, erythrocytes, and platelets [58], suggesting different kinetics for handling of EPA and DHA. It is unclear why these two n-3 PUFAs show different kinetics, but the slower incorporation of DHA than EPA could be a mechanism to prevent a too rapid change in membrane fluidity with increased DHA availability. Browning et al. [56] demonstrated a clear dose-dependent incorporation of EPA and DHA into blood lipids and blood cells, and it is likely that this occurs for skeletal muscle also, but this has not been reported yet in humans.

McGlory et al. [57] linked the progressive increase in n-3 PUFAs in muscle over time with changes in levels of certain proteins involved in anabolic signalling in the muscle. They demonstrated a time-dependent increase in protein tyrosine kinase 2 which was higher after 4 weeks than at baseline and modest, though not significant, time-dependent increases in ribosomal protein S6 kinase beta-1 (P70S6K) and in eukaryotic translation initiation factor 4E-binding protein 1. mTOR was higher at week 2 than at baseline. These effects suggest that n-3 PUFAs acids could promote MPS. This was explored in two studies by Smith et al. [56, 59]. In the first of these studies [59], daily supplementation with 1.86-g EPA and 1.5-g DHA for 8 weeks in healthy older adults (age > 65 years) increased the rate of MPS that occurred in response to a hyperaminoacidemic–hyperinsulinemic clamp. The increase was about 240% above the rate observed with the clamp at study entry but did not occur in the absence of the clamp. EPA and DHA increased the serine 2448 phosphorylation of mTOR and the threonine 369 phosphorylation of P70S6K seen in response to the clamp. In a similar study conducted in younger adults [56], protein synthesis during the clamp was increased by about 45% compared to baseline by the n-3 PUFAs. The clamp increased serine 2448 phosphorylation of mTOR, threonine 369 phosphorylation of P70S6K, and threonine 208 phosphorylation of protein kinase B (also known as AKT), but the phosphorylation of these signalling proteins in response to the clamp was greater after the period of n-3 PUFA intake compared with study entry. Thus, these studies provide evidence that bioactive n-3 PUFAs significantly increase the muscle protein anabolic response to a hyperaminoacidemic–hyperinsulinemic clamp in young and middle aged and in older adults, and they link this metabolic action to altered activation of proteins involved in the signalling linking amino acids and insulin to the pathway of protein synthesis. The n-3 PUFAs did not affect the basal rate of protein synthesis from amino acids, so their action seems to be linked to augmenting the anabolic effect of other signals. McGlory et al. [60] reported that supplementation of young adult females with 2.97-g EPA and 2.03-g DHA daily resulted in better maintenance of quadriceps volume during 2 weeks of leg immobilization and that this was linked to retention of a higher rate of myofibrillar protein synthesis during the immobilization period. Most recently, Engelen et al. [61] showed an EPA plus DHA dose-dependent reduction of whole-body protein breakdown during the postabsorptive period and an EPA plus DHA dose-dependent increase in whole-body protein synthesis during the feeding period in patients with chronic obstructive pulmonary disease. Taken together, these human studies indicate that EPA and DHA alter the expression and activation of anabolic signalling proteins in human skeletal muscle, decrease MPB, and increase MPS. These effects are of relevance to situations where there is risk of loss of muscle mass due to ageing, disuse, or disease. In this context, a recent meta-analysis of human studies in heterogeneous population groups identified that lean mass and skeletal muscle mass are favoured by higher intakes of EPA and DHA [62].

Enhancing endogenous SPM production by providing n-3 PUFAs early in the course of disease might become an important strategy. In the context of gastrointestinal surgery, SPMs are especially relevant in anastomotic healing through their influence on the polarization of macrophages [63–65]. The evidence for SPMs being important is infections which are manifested in direct and indirect mechanisms in both viral and bacterial infections.

SPMs have many other clinically relevant functions for the perioperative period including antimicrobial activity via several well-described mechanisms (64). Additionally, they reduce biofilm formation and the secretion of exotoxins such as pyocyanin, and they also potentiate antibiotic function which lowers antibiotic requirements [66]. Inflammatory bowel disease would appear to be an obvious target for the use of SPMs. Resolvin E1 has recently been shown to promote healing in intestinal epithelial wounds [67]. In the acute and chronic inflammation induced by COVID-19, SPMs have been shown to limit excessive, uncontrolled inflammation and limit collateral damage [68].

Recent work evaluating SPMs in metabolism has shown that resolvin E3 specifically ameliorates diet-induced insulin resistance [69]. SPMs can also improve muscle metabolism in various disease states. Castor-Macias et al. have shown in a mammalian model that maresin-1 (made from DHA) improves muscle regeneration and myogenesis after volumetric muscle tissue loss. Maresin-1 also resulted in increased strength with decreased fibrosis [70]. Recent work by McClain et al. has shown that resolvin D1 significantly decreased hepatic injury in an ethanol/lipopolysaccharide model. Interestingly, this benefit appears very stereospecific as there was no benefit with resolvin E1 [71].

## Conclusion

Medical nutrition therapy must be individualized and adapted to patient’s evolving needs and the clinical condition. Muscle catabolism is present from admission in critically ill patients, leading to loss of muscle mass and its adverse clinical impacts. Existing approaches include progressively increasing medical nutrition therapy and providing adequate protein, with recent studies suggesting that protein provision should be gradually increased over time and based on patient stability. Unresolved inflammation may be a key factor in driving persistent muscle catabolism. Control of inflammatory processes may be the key to the effectiveness of nutritional support, especially now that it has been demonstrated that inflammation resolution is an active process. The n-3 fatty acids EPA and DHA are substrates for synthesis of mediators termed SPMs that act to resolve inflammation. There is evidence from non-critical care settings that oral doses of EPA + DHA in the range of 2.5–5 g/day increase muscle protein synthesis, decrease muscle protein breakdown, and preserve muscle mass. Supply of EPA and DHA by the parenteral route enables higher doses to be delivered, and this might induce higher incorporation into target cells and tissues (e.g. skeletal muscle), and this might induce greater and faster effects. SPMs may be responsible for some of the effects of EPA and DHA, especially upon muscle protein breakdown. Given these findings, provision of EPA and DHA at nutritional doses as part of nutrition therapy in combination with moderate protein doses seems to be a strategy to prevent the persistence of inflammation, the related anabolic resistance, and muscle loss. Thus, the combination of protein and n-3 PUFAs might have a greater physiological and clinical impact than either one alone but this has not been examined. It will be important to test this idea, perhaps using a 2 x 2 factorial study design in relevant target groups such as the elderly and those at risk of muscle loss through disease or immobilization.

## Data Availability

Not applicable as there was no use of data sets.

## Abbreviations

CRP: C-reactive proteins
EPA: Eicosapentaenoic acid
DHA: Docosahexaenoic acid
mTOR: Mammalian target of rapamycin receptor
n-3 PUFA: Omega-3 polyunsaturated fatty acid
SPMs: Specialized pro-resolving mediators
